# Protein and Energy Intake Assessment and Their Association With In-Hospital Mortality in Critically Ill COVID-19 Patients: A Prospective Cohort Study

**DOI:** 10.3389/fnut.2021.708271

**Published:** 2021-08-19

**Authors:** Melika Hajimohammadebrahim-Ketabforoush, Zahra Vahdat Shariatpanahi, Maryam Vahdat Shariatpanahi, Erfan Shahbazi, Shaahin Shahbazi

**Affiliations:** ^1^Department of Clinical Nutrition and Dietetics, Faculty of Nutrition Sciences and Food Technology, National Nutrition and Food Technology Research Institute, Shahid Beheshti University of Medical Sciences, Tehran, Iran; ^2^Department of Psychiatry, Faculty of Medicine, Azad University of Medical Sciences, Tehran, Iran; ^3^Department of Food Science, Faculty of Nutrition and Food Technology, National Nutrition and Food Technology Research Institute, Shahid Beheshti University of Medical Sciences, Tehran, Iran; ^4^Department of Gastroenterology, Faculty of Medicine, Ilam University of Medical Sciences, Ilam, Iran

**Keywords:** COVID-19, protein, energy, nutritional intake, ICU, mortality

## Abstract

**Background and Aim:** It is partially known that nutritional intake could alleviate proteolysis and course of disease severity in patients with COVID-19; however, not enough data are available in this regard. The present study aimed to assess protein and energy intake and their association with in-hospital mortality in critically ill COVID-19 patients.

**Methods:** A total of 126 patients with COVID-19, who were critically ill, ≥5 days and a subset of 111 patients in ICU ≥10 days completed the present prospective observational cohort study.

**Results:** Protein and energy intakes on Day 5 of ICU admission in survivors were 46 and 58% of target values, respectively. These values in non-survivors were 42 and 50% of target values, respectively (*p* < 0.05). In the sample ≥10 days, protein and energy intakes in survivors reached 64 and 87% of target values, respectively, without statistically significant differences with non-survivors. In the sample ≥5 days, Cox proportional hazard regression was adjusted for GLIM, APACHE II, comorbidity, and age; the results indicated that the patients with protein and energy intake lower than 0.59 g/kg/day and 14 kcal/kg/day, had ~2-fold mortality hazard (protein: HR, 2.38; 95% CI, 1.40–4.03; *P* = 0.001 and energy: HR, 2.23; 95% CI, 1.27–3.92; *P* = 0.005).

**Conclusion:** Actual protein and energy intakes in critically ill patients with COVID-19 are in suboptimal levels compared with goal recommendations in these patients. Moreover, higher amounts of protein and energy intakes in the early acute phase were significantly associated with better survival and lower risk of in-hospital mortality.

## Introduction

The current terrible pandemic (coronavirus disease 2019) created by the severe acute respiratory syndrome coronavirus 2 (SARS-CoV-2) has affected a huge number of people. The victims experience mild to critical degrees of infection (1, 2). Up to 30% of infected patients present with an acute respiratory distress syndrome (ARDS) require urgent respiratory and hemodynamic support in the intensive care unit (ICU) (3). A highlighted feature in these catabolic patients with coronavirus disease 2019 (COVID-19) is the cytokine storm that could result in muscle breakdown and proteolysis (4). In several previous studies on non-COVID critically ill, muscle breakdown was reported to be associated with worsened clinical outcomes (5–7). Severe malnutrition and muscle mass losses could both originate and lead to COVID-19 severity and are known as risk factors in mortality (8–10). Therefore, nutritional assessments are known to be a pivotal component of standard care for patients with COVID-19, which should be included in the applied therapeutic strategy (9, 10). To date, however, no studies have examined protein and energy intake and their association with in-hospital mortality in critically ill patients with COVID-19; consequently, it is not well understood whether survival benefits from the amount of protein and energy intake depend on the malnourished state of the patient. Certain studies on patients with other critical illness hospitalized in ICU have shown beneficial effects of greater protein (11, 12) and energy intake (13, 14) on various adverse clinical outcomes, yet other research have not reported any benefits (15). There are other studies suggesting the harmful effects of increased delivery of protein (16) and energy (17) in critically ill patients. Therefore, considering these controversial results, the scarce-related studies on patients with COVID-19, and evaluation of the outcomes during ICU stay and hospitalization, we conducted the current study to assess protein and energy intake and investigate their association with in-hospital mortality in critically ill patients with COVID-19.

## Materials and Methods

The current prospective cohort study was conducted from August, 2020, to March, 2021, in a university hospital. The study was approved by the ethics committee of the university. Adult patients ≥18 years old with positive real-time fluorescence polymerase chain reaction (RT-PCR) for COVID-19, who were critically ill, were included. Informed consent was obtained from the subjects or their surrogates. Patients who are critically ill are defined as cases with respiratory failure, shock, or multiorgan dysfunction, who should be treated in the intensive care unit (ICU) according to World Health Organization (WHO) classification (18). Some of the patients from our previous cohort study were included in the current study (19). The exclusion criteria comprised the patients without weight and height data, participation in other clinical trials, pregnancy, ICU stay <72 h, and those with end-stage kidney disease, cirrhosis, and cancer. At admission, the nutritional assessment was carried out, using the Global Leadership Initiative on Malnutrition (GLIM) criteria. Weight and height were not possible to measure in most patients; therefore, we were satisfied with self-report by the patients or their caregivers. BMI was calculated as weight in kilograms divided by squared height in meters. During hospitalization in the ICU, data on food and formula intakes were recorded for all the subjects. In the patients who were on oral route feeding, a trained nurse collected the food record data. In those who were on nutritional support, either enteral or parenteral nutrition, intake-related data were obtained from medical records. Based on the guidelines on protein and energy requirement in patients with COVID-19 in the ICU (20), we considered 1.3 g/kg/day and 25 kcal/kg/day as protein and energy targets, respectively. Moreover, if the patients had BMI < 30, the actual body weight was considered for protein and energy calculations. Meanwhile, for BMI ≥ 30, we referred to the ideal body weight by multiplying the height of the patient by a BMI of 25. The mean ratio of actual protein and energy intakes to the target protein and energy intakes during 5 and 10 days from ICU admission was measured in order to calculate protein and energy intake ratios (%). Based on the GLIM criteria, the nutritional assessment was performed for all the subjects to be included in malnourished and well-nourished groups. GLIM is a two-step approach defined by the association of one phenotypic criterion (non-volitional weight loss, low BMI) and one etiologic criterion (reduced food intake or assimilation, disease burden/inflammatory condition) (21). The investigated clinical outcome in the present study included in-hospital mortality.

### Statistical Analysis

SPSS software version 22.0 was employed to analyze the data (Statistical Package for the Social Sciences, IBM Corp., Armonk, New York, USA). To find the differences between the alive and dead groups concerning normal and abnormal distributed variables, Student's *t*-test and Mann–Whitney *U* test were used, respectively. The results are reported as mean ± standard deviation for parametric tests and as median (Q1–Q3) for nonparametric tests. Chi-square or Fisher's exact tests were utilized to evaluate the differences in the distribution of categorical variables. The Cox proportional hazards models were used to estimate the hazard of mortality with protein and energy intake with and without adjustments for GLIM, APACHE II score, comorbidity presence, and age. We applied Kaplan–Meier curves and the log rank test for estimation and comparison of crude survival between the patients in the two categories of protein and energy intake and GLIM criteria. Based on GLIM criteria, the patients were categorized into well-nourished and malnourished patients. Moreover, based on protein and energy, they were categorized into two groups as lower and higher than the median of protein and energy intake ratios. In the Cox models, to estimate the hazard of mortality, actual protein (g/kg/day) and energy (kcal/kg/day) intakes were used, in which the patients were categorized into two groups based on their median intakes on days 5 and 10 separately. In all the analyses, *p* < 0.05 was considered to be statistically significant.

## Results

**Figure 1:**
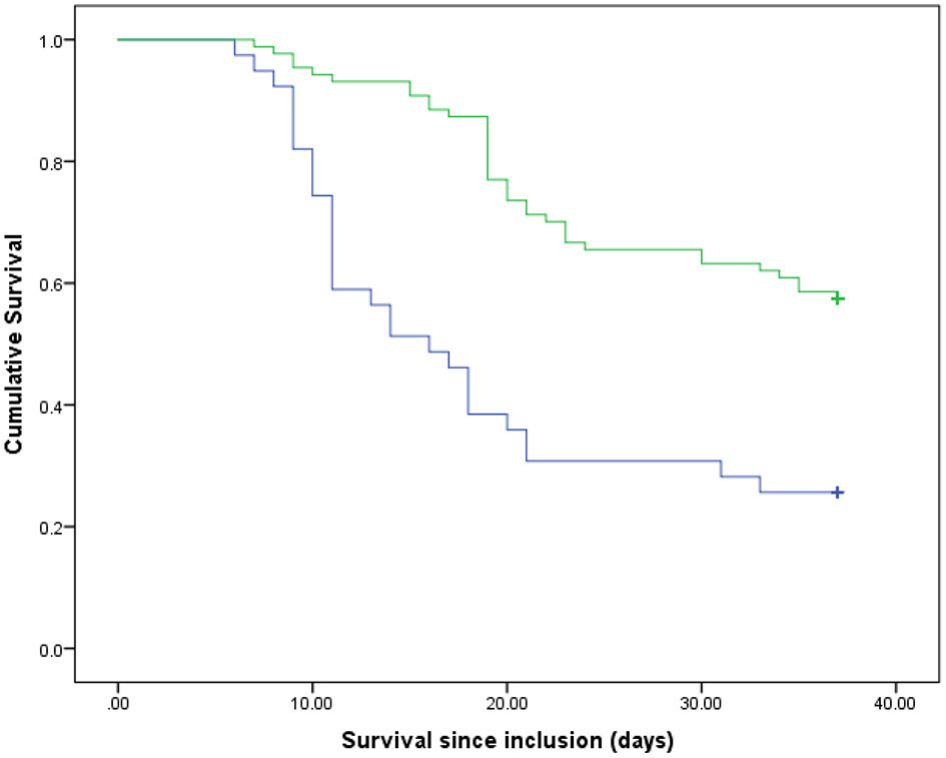
Survival rates of the participants compared between two groups of the protein intake ratio. Blue line, patients with lower than median of the protein intake ratio; green line, patients with higher than median of the protein intake ratio. *p* < 0.001 (log rank).

**Figure 2:**
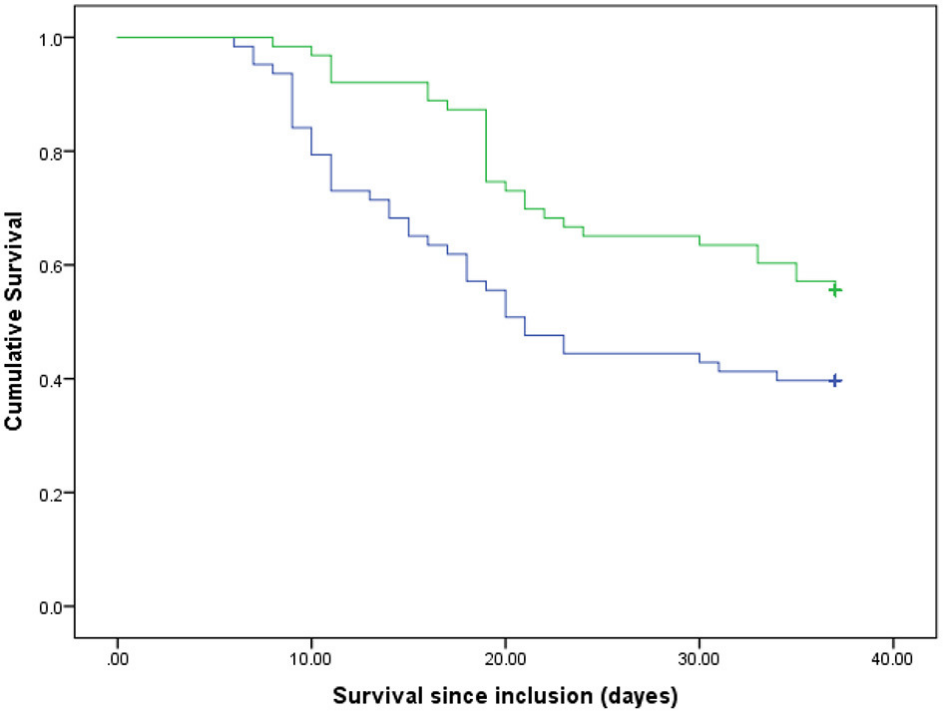
Survival rates of the participants compared between two groups of the energy intake ratio. Blue line, patients with lower than median of the energy intake ratio; green line, patients with higher than median of the energy intake ratio. *P* = 0.015 (log rank).

**Figure 3:**
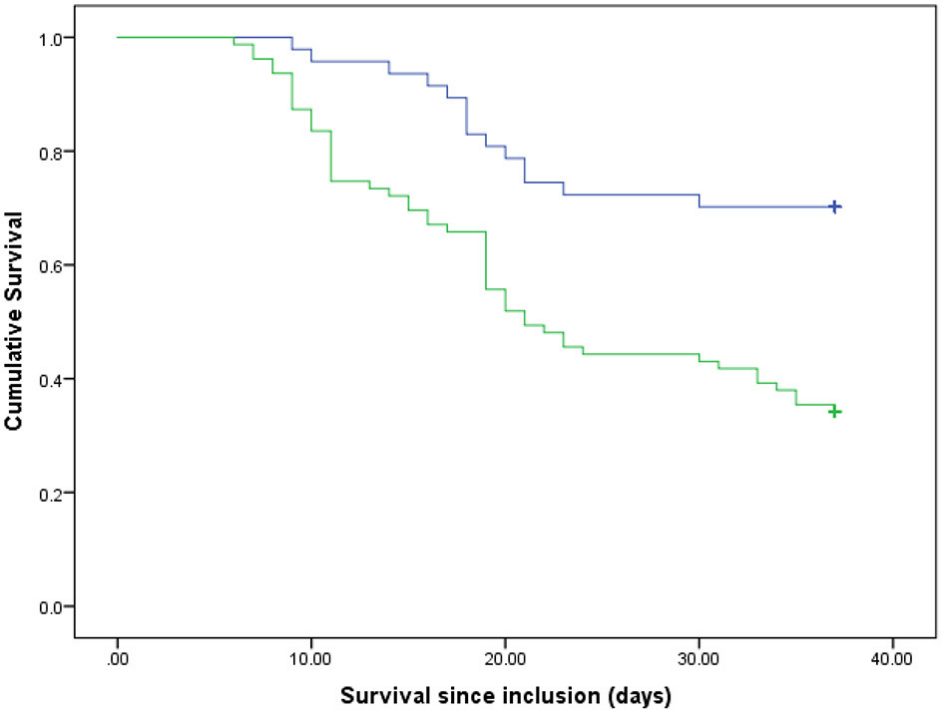
Survival rates of the participants based on GLIM criteria. Blue line, well-nourished patients; green line, malnourished patients. *p* < 0.001 (log rank).

Out of the total of critically ill patients with COVID-19 (*n* = 126) during the time frame of the present study (37 days), 60 survived and 66 died. The mean ± SD ages of the survivors and non-survivors were 57.85 ± 11.86 and 62.36 ± 15.08, respectively (*P* = 0.06). Table 1 represents the baseline clinical characteristics and nutritional intakes of the patients with survival status. As could be seen, protein and energy intakes on day 5 of ICU admission in the survivors were respectively .59 ± 0.11 g/kg/day and 14.40 ± 3.53 kcal/kg/day; these values were ~46 and 58% of target values, respectively. Protein and energy intakes on day 5 of ICU admission in the non-survivors were .54 ± 0.11 g/kg/day and 12.55 ± 2.97 kcal/kg/day, which were approximately 42 and 50% of target values, respectively. These differences in comparison with the survivors were statistically significant (Protein: *P* = 0.015; Energy, *P* = 0.002). By day 10 of ICU admission, 15 patients had died; therefore, they were excluded in day 10 of the analysis. In the patients who were hospitalized in ICU longer than 10 days (*n* = 111), protein and energy intakes in the survivors were .82 ± 0.12 g/kg/day and 21.81 ± 4.19 kcal/kg/day, which were approximately 64 and 87% of target values, respectively. Protein and energy intakes on day 10 of ICU admission in the non-survivors (*n* = 51) were .79 ± 0.12 g/kg/day and 20.99 ± 3.83 kcal/kg/day, respectively; these values were ~61 and 84% of target values. However, these differences in comparison with the survivors were not statistically significant (Table 1). In order to estimate mortality risk and its correlation with confounders, some parameters are indicated in Table 1, which seems to have potential predictive value on mortality hazard with differences between the survivors and non-survivors at the level of 0.15, included in Cox proportional hazards models. Primarily, the crude survival was shown through Kaplan-Meier curves. In all the patients, regardless of how long they stay in ICU, the median of protein and energy intake ratios was 60% (50.97–60) and 86.24% (73.11–95.42), respectively. As shown in Figure 1, the survival time of the patients with a lower-than-median-protein-intake ratio was significantly shorter than those with a higher-than-median-protein-intake ratio [19.92 days (16.32–23.51) vs. 29.94 days (27.89–31.99), *P* < 0.001; log rank]. Furthermore, the median of survival in the patients with a lower-than-median-energy-intake ratio was 23.82 days (20.87–26.77), while in those with a higher-energy-intake ratio was 29.85 (27.45–32.25), *P* = 0.015; log rank (Figure 2). When the patients were generally categorized based on GLIM criteria into two different categories as well-nourished and malnourished, Kaplan–Meier curve showed that survival was better in the well-nourished patients than that in the malnourished [31.38 (28.79–33.79) vs. 24.13 days (21.57–26.70), *P* < 0.001; log rank] (Figure 3). In Cox proportional hazards models, the patients were separated based on their stay time in ICU into a subject that survived more than 5 and 10 days. As depicted in Table 2, in the sample ≥5 days, the patients with protein intake (g/kg/day) lower than median relative to those with higher-than-median protein intake had 2.9-fold mortality hazard (*p* < 0.0001). After adjusting the model for GLIM, APACHE II, comorbidity presence, and age, this relationship remained significant (*P* = 0.001). Moreover, the subjects with energy intake (kcal/kg/day) lower than median relative to those with higher-than-median energy intake had nearly 2-fold mortality hazard (*P* = 0.04). After the model was adjusted for GLIM, APACHE II, comorbidity presence, and age, this relationship remained significant (*P* = 0.005). However, in the sample ≥10 days, after full adjustment of the model run, these correlations became statistically insignificant (Table 2).

**Table 1:** Baseline and clinical characteristics of patients.

**Variable**	**Total** **(***n*** = 126)**	**Survivors** **(***n*** = 60)**	**Non-survivors** **(***n*** = 66)**	***P*** **Value**
Age	60.21 ± 13.78	57.85 ± 11.86	62.36 ± 15.08	0.06
Female, *n* (%)	61 (48)	27 (45%)	34 (52%)	0.46
Days from illness onset to admission,	8 (7-9)	8 (7-9)	8 (7-9)	0.88
APACHE II	15.73 ± 3.36	15.23 ± 3.07	16.20 ± 3.65	0.06
Weight (Kg)	68.13 ± 13.86	67.36 ± 13.37	68.83 ± 14.36	0.55
O_2_ Therapy, *n* (%)				0.11
HFNC	15 (12)	9 (15%)	6 (9%)	
NIV	40 (32)	23 (38%)	17 (26%)	
MV	71 (56)	28 (47%)	43 (65%)	
Comorbidity, *n* (%)	52 (41)	17 (28%)	35 (53%)	0.005
Medication, *n* (%)				
Antiviral	126 (100)	60 (100)	66 (100)	1
Antibiotic	64 (51)	21 (35)	43 (65)	0.001
Glucocorticoid	126 (100)	60 (100)	66 (100)	1
Malnutrition (GLIM)	76 (63)	27 (45%)	52 (79%)	0.001
Actual protein intakeon day 5 (g/kg/day)	0.57 ± 0.11	0.59 ± 0.11	0.54 ± 0.11	0.015
Protein intake ratio on day 5 (%)	43.95 ± 8.90	45.96 ± 8.64	42.11 ± 8.80	0.015
Actual protein intakeon day 10^*^ (g/kg/day)	0.81 ± 0.12	0.82 ± 0.12	0.79 ± 0.12	0.162
Protein intake ratio on day 10^*^ (%)	62.56 ± 9.33	63.71 ± 9.25	61.22 ± 9.34	0.162
Actual energy intakeon day 5 (Kcal/kg/day)	13.43 ± 3.37	14.40 ± 3.53	12.55 ± 2.97	0.002
Energy intake ratio on day 5 (%)	53.74 ± 13.49	57.62 ± 14.15	50.22 ± 11.90	0.002
Actual energy intakeon day 10^*^ (Kcal/kg/day)	21.43 ± 4.04	21.81 ± 4.19	20.99 ± 3.83	0.291
Energy intake ratio on day 10^*^ (%)	85.75 ± 16.16	87.25 ± 16.79	83.99 ± 15.35	0.291

*APACHE, acute physiology and chronic health evaluation; HFNC, high flow nasal cannula; NIV, noninvasive ventilation; MV, mechanical ventilation; GLIM, global leadership initiative on malnutritio*.

* * The sample size in the non-survivals on day 10 was 51 patients because 15 patients had died by day 10*.

**Table 2:** Protein and energy intake as a predictor of in-hospital mortality in critically ill patients with COVID-19.

	**Sample in ICU** **≥** **5 days**	
**Outcome**	**Protein intake (g/kg/day)** ^***a***^	**Energy intake (kcal/kg/day)** ^***b***^
Mortality ^*c*^	2.958 (1.768–4.950)^*^	1.516 (1.933–2.462)^**^
Adjusted ^*d*^	2.385 (1.409–4.037)^**^	2.239 (1.278–3.920)^**^
	**Sample in ICU** **≥** **10 days**	
**Outcome**	**Protein intake (g/kg/day)** ^***a***^	**Energy intake (kcal/kg/day)** ^***b***^
Mortality ^*c*^	1.452 (0.838–2.516)	1.287 (0.743–2.231)
Adjusted ^*d*^	1.718 (0.932–3.167)	1.718 (0.884–3.340)

a, b *Protein and energy intakes categorized to two groups based on their median, and the groups higher than median were considered as reference categories*.

c *Evaluated by the Cox proportional hazards model with an outcome of mortality by day 37 after ICU admission*.

d *Adjusted for GLIM, APACHE II, comorbidity presence, and age*.

* *p value significant at the 0.0001 level*.

** *p value significant at the 0.05 level*.

## Discussion

In general, in the present study on the critically ill patients with COVID-19, who were hospitalized in ICU during 37 days, actual protein and energy intakes were approximately 46 and 58% of the recommended target values, respectively, in the survivors on day 5 from admission. These percentages reached 64 and 87% of target values on day 10. In the patients who died, these percentages were lower. The crude survival estimated with Kaplan–Meier analysis demonstrated that the patients with protein and energy intakes ratios lower than 60 and 86% of target values, respectively, had lower survival time than those with amounts higher than these cutoffs. Moreover, survival was better in well-nourished patients than that in the malnourished ones. Furthermore, the results from Cox proportional hazards models in the sample ≥5 days indicated that the subjects with actual protein and energy intake, respectively, lower than .59 g/kg/day and 14 kcal/kg/day had near to ~ 2-fold mortality hazard compared with those with higher cutoffs. However, in the sample ≥10 days, after fully adjusting the model run, these correlations became statistically insignificant. Recently, a study by O'Sullivan et al. (22) has shown that critical care patients with COVID-19 had protein and energy intakes of 44.2 and 69.8% of target estimated requirements in the early acute phase, respectively. These values reached 67.8 and 81.5% target estimated requirements in the late acute phase. Additionally, Yue et al. (23) reported the average of 15.3 kcal/kg/day for actual energy intake and 0.62 g/kg/day for actual protein intake in critically ill patients with COVID-19 for duration of 7 days. The results of our study are closely consistent with these mentioned studies. In the patients who died in our study, these percentages were significantly lower than those in the survivors, which could be justified given the worsened overall condition and GI intolerance of the patient (22). Even though some of these studies have assessed the nutritional intake in critically ill patients with COVID-19, its association with in-hospital mortality remains extremely unclear. There are available data on patients with other critical illnesses hospitalized in the ICU, who showed greater protein (11, 12) and energy (13, 14) intake correlated with alleviated adverse clinical outcomes. Nicolo et al. (12) indicated that in a critically ill population, mean protein and energy intake for the 4-day sample were 60.5 and 64.1% of the prescribed amount, respectively. These amounts reached 66.7 and 70.7% of the prescribed quantity for the 12-day sample. Their study concluded that achieving at least 80% of prescribed protein intake may be important for better survival and shorter hospitalization in patients in the ICU. They did not find this correlation for energy intake. However, another study reported that, with the increase of 1,000 cal energy intake per day, 60-day mortality decreased statistically significantly in patients who were critically ill. This correlation was also observed for protein intake (13). Arabi et al. (24), in a *post hoc* analysis, showed that, among 729 mixed patients who were critically ill, there were no significant differences between the patients with higher protein intake (average 0.8 ± 0.3 g/kg/day) and those with lower-protein intake (average 0.6 ± 0.2 g/kg/day) in terms of 90-day mortality risk. Certain studies suggested that early high-protein intake during and up to 4 days from ICU admission in patients with no COVID-19 was associated with low mortality (11, 25, 26) while, due to the possibility of refeeding syndrome, energy overfeeding is harmful (11). Nevertheless, in their study, the level of energy intake was considerably beyond that delivered to the patients in the current study. In comparison with other studies on critically ill settings, in which protein and energy cutoffs were more than in the present study, these cutoff values were lower (22, 23), which is consistent with a few of the same studies on patients with COVID-19. Thus, it is obvious that even negligible differences between these little intakes could significantly alter the hazard of mortality. One of the main reasons for this lower intake in patients with COVID-19 than other patients who were critically ill could be attributed to nutrition and the panic of health care providers of the viral transmission due to close contact with the patients. On the other hand, as described in the studies mentioned, there is no agreement on the optimal amount of protein and energy intake in patients who are critically ill; therefore, further studies are necessary, particularly on critically ill patients with COVID-19. One possible reason why some studies have not found any survival benefits of higher protein and energy intake could be the measurement of average protein and energy intake during hospitalization rather than separating early and late acute phases. In our study, we found meaningful survival benefits of higher protein and energy intakes in the early acute phase and not in the late acute phase. It may be linked to the fact that, if nutritional support in the acute phase was postponed, other factors, such as medical conditions, comorbidity, worsening breath, and cytokine storm, became more significant, which influenced death occurrence. Therefore, time is a key component of nutritional survival benefits (26). Furthermore, Compher et al. (27), in a large, diverse sample of mixed patients who were critically ill, indicated that lower mortality and shorter time to discharge alive were associated with greater protein and energy intake in the patients with a high NUTRIC score in both 4- and 12-day samples. However, according to the new guideline from the global clinical nutrition community (21, 28), Global Leadership Initiative on Malnutrition (GLIM) criteria are better tools to diagnose malnutrition in patients with COVID-19. Therefore, we categorized our patients, based on these criteria, to well-nourished and malnourished patients. Consistent with some previous studies (8–10), we also found that malnutrition is known as a risk factor in mortality in critically ill patients with COVID-19. Meanwhile, due to our study with a small sample size, we could not stratify analysis to high and low nutritional risk to evaluate the survival status, such as what Compher et al. (27) were doing. Specifically, in the present study, the number of patients in the sample ≥10 days was falling because 15 patients died up to day 10. If the sample size was bigger, it would also be possible to see significant survival benefits from protein and energy intakes in the sample ≥10 days. In our analysis of a prospective cohort study, lower in-hospital mortality was associated with greater early protein and energy intake in critically ill patients with COVID-19. However, the current study had some limitations. Our study was single-centered; therefore, it is not possible to generalize our results. This study suggested that more successful delivery of goal protein and energy intake is associated with the strongest survival in the critically ill patients with COVID-19. However, further studies with larger sample sizes on more diverse corona centers are required.

## Conclusions

In conclusion, our findings revealed that the actual protein and energy intakes in critically ill patients with COVID-19 hospitalized in ICU are at suboptimal levels. Hence, it could be recommended that these amounts are in further agreement with the guidelines, which stated goal amounts of protein and energy intakes in the category of these patients who were critical. Moreover, higher amounts of protein and energy intakes in the early acute phase were significantly associated with better survival and a lower risk of in-hospital mortality. Future clinical trials should determine the optimal levels of protein and energy intake in the early vs. the late acute phase in critically ill patients with COVID-19.

## Data Availability Statement

The raw data supporting the conclusions of this article will be made available by the authors, without undue reservation.

## Ethics Statement

The studies involving human participants were reviewed and approved by Ilam University of Medical Sciences Ethics Committee. The patients/participants provided their written informed consent to participate in this study.

## Author Contributions

Material preparation, data collection, and analysis were performed by SS, ZV, MH-K, MV, and ES. The first draft of the manuscript was written by MH-K and ZV. All authors contributed to the study conception and design, commented on previous versions of the manuscript, and read and approved the final manuscript.

## Conflict of Interest

The authors declare that the research was conducted in the absence of any commercial or financial relationships that could be construed as a potential conflict of interest.

## Publisher's Note

All claims expressed in this article are solely those of the authors and do not necessarily represent those of their affiliated organizations, or those of the publisher, the editors and the reviewers. Any product that may be evaluated in this article, or claim that may be made by its manufacturer, is not guaranteed or endorsed by the publisher.

## Abbreviations

APACHE: acute physiology and chronic health evaluation
ARDS: acute respiratory distress syndrome
BMI: body mass index
GLIM: global leadership initiative on malnutrition
ICU: intensive care unit
RT-PCR: real-time fluorescence polymerase chain reaction
SARS-CoV-2: severe acute respiratory syndrome coronavirus
SGA: subjective global assessment
WHO: World Health Organization.

## References

[1] Zhu N Zhang D Wang W Li X Yang B Song J. A novel coronavirus from patients with pneumonia in China, 2019. N Engl J Med. (2020) 382:727–33. 10.1056/NEJMoa2001017 31978945 PMC7092803

[2] Zhao X Li Y Ge Y Shi Y Lv P Zhang J. Evaluation of nutrition risk and its association with mortality risk in severely and critically Ill COVID-19 patients. JPEN J Parenter Enteral Nutr. (2020) 45:32–42. 10.1002/jpen.1953 32613660 PMC7361906

[3] Huang C Wang Y Li X Ren L Zhao j Hu Y. Clinical features of patients infected with 2019 novel coronavirus in Wuhan, China. Lancet. (2020) 395:497–506. 10.1016/S0140-6736(20)30183-5 31986264 PMC7159299

[4] Uribarri J Shamy OE Sharma S Winston J. COVID-19-Associated acute kidney injury and quantified protein catabolic rate: a likely effect of cytokine storm on muscle protein breakdown. Kidney Med. (2021) 3:60–3. 10.1016/j.xkme.2020.09.011 33283182 PMC7706425

[5] Koukourikos K Tsaloglidou A Kourkouta L. Muscle atrophy in intensive care unit patients. Acta Inform Med. (2014) 22:406–10. 10.5455/aim.2014.22.406-410 25684851 PMC4315632

[6] Nakanishi N Tsutsumi R Okayama Y Takashima T Ueno Y Itagaki T. Monitoring of muscle mass in critically ill patients: comparison of ultrasound and two bioelectrical impedance analysis devices. J Intensive Care. (2019) 7:61. 10.1186/s40560-019-0416-y 31890223 PMC6916000

[7] Puthucheary ZA Rawal J McPhail M Connolly B Ratnayake G Chan P. Acute skeletal muscle wasting in critical illness. JAMA. (2013) 310:1591–600. 10.1001/jama.2013.278481 24108501

[8] Mertens E Peñalvo JL. The burden of malnutrition and fatal COVID-19: a global burden of disease analysis. Front Nutr. (2021) 7:619850. 10.3389/fnut.2020.619850 33553234 PMC7858665

[9] Thibault R Seguin P Tamion F Pichard C Singer P. Nutrition of the COVID-19 patient in the intensive care unit (ICU): a practical guidance. Crit Care. (2020) 24:447. 10.1186/s13054-020-03159-z 32684170 PMC7369442

[10] Thibault R Coëffier M Joly F Bohé J Schneider SM Déchelotte P. How the -19 epidemic is challenging our practice in clinical nutrition-feedback from the field. Eur J Clin Nutr. (2020) 75:407–16. 10.1038/s41430-020-00757-6 32939042 PMC7492685

[11] Weijs PJ Looijaard WG Beishuizen A Girbes A Oudemans-van Straaten HM. Early high protein intake is associated with low mortality and energy overfeeding with high mortality in non-septic mechanically ventilated critically ill patients. Crit Care. (2014) 18:701. 10.1186/s13054-014-0701-z 25499096 PMC4279460

[12] Nicolo M Heyland DK Chittams J Sammarco T Compher C. Clinical outcomes related to protein delivery in a critically ill population: A multicenter, multinational observation study. JPEN J Parenter Enteral Nutr. (2016) 40:45–51. 10.1177/0148607115583675 25900319

[13] Alberda C Gramlich L Jones N. The relationship between nutritional intake and clinical outcomes in critically ill patients: Results of an international multicenter observational study. Intensive Care Med. (2009) 35:1728–37. 10.1007/s00134-009-1567-4 19572118

[14] Elke G Kuhnt E Ragaller M Frerichs I FM Löffler M German Competence Network Sepsis (SepNet): Enteral nutrition is associated with improved outcome in patients with severe sepsis. A secondary analysis of the VISEP trial. Med Klin Intensivmed Notfmed. (2013) 108:223–33. 10.1007/s00063-013-0224-4 23455443

[15] Arabi YM Aldawood AS Haddad SH Tamim H Haddad S Jones G. PermiT Trial Group: Permissive underfeeding or standard enteral feeding in critically ill adults. N Engl J Med. (2015) 372:2398–408. 10.1056/NEJMoa1502826 25992505

[16] Casaer MP Wilmer A Hermans G Wouters P Mesotten D Van den Berghe G. Role of disease and macronutrient dose in the randomized controlled EPaNIC trial: A post hoc analysis. Am J Respir Crit Care Med. (2013) 187:247–55. 10.1164/rccm.201206-0999OC 23204255

[17] Braunschweig CA Sheean PM Peterson SJ Perez S Freels S Lateef O. Intensive nutrition in acute lung injury: A clinical trial (INTACT). JPEN J Parenter Enteral Nutr. (2015) 39:13–20. 10.1177/0148607114528541 24722769 PMC4205213

[18] World Health Organization. Clinical management of COVID-19: interim guidance, 27 May 2020. World Health Organization. (2020) Available online at: https://apps.who.int/iris/handle/10665/332196

[19] Shahbazi S Hajimohammadebrahim-Ketabforoush M Vahdat Shariatpanahi M Shahbaz E Vahdat Shariatpanahi Z. The validity of the global leadership initiative on malnutrition criteria for diagnosing malnutrition in critically ill patients with COVID-19: A prospective cohort study. Clin Nutr ESPEN. (2021) 43:377–82. 10.1016/j.clnesp.2021.03.020 34024543 PMC8015411

[20] Minnelli N Gibbs L Larrivee J Sahu KK. Challenges of Maintaining Optimal Nutrition Status in COVID-19 Patients in Intensive Care Settings. JPEN J Parenter Enteral Nutr. (2020) 44:1439–46. 10.1002/jpen.1996 32799322 PMC7461277

[21] Cederholm T Jensen GL Correia MITD Gonzalez MC Fukushima R Higashiguchi T. GLIM criteria for the diagnosis of malnutrition - A consensus report from the global clinical nutrition community. Clin Nutr Elsevier Ltd. (2019) 38:1–9. 10.1016/j.clnu.2018.08.002 30181091

[22] O'Sullivan E McMorrow A McCormack D O'Connor E. Analysis of nutrition support in covid19 critical care patients. Clin Nutr ESPEN. (2020) 40:412–690. 10.1016/j.clnesp.2020.09.684

[23] Yue X Li M Wang Y Zhang J Wang X Kan L. Nutritional Support and Clinical Outcome of Severe and Critical Patients With COVID-19 Pneumonia. Front Nutr. (2020) 7:581679. 10.3389/fnut.2020.581679 33330582 PMC7711882

[24] Arabi YM Al-Dorzi HM Mehta S Tamim HM Haddad SH Jones G. Association of protein intake with the outcomes of critically ill patients: a post hoc analysis of the PermiT trial. Am J Clin Nutr. (2018) 108:988–96. 10.1093/ajcn/nqy189 30475959

[25] Looijaard WGPM Dekker IM Beishuizen A Girbes ARJ. Oudemans-van Straaten HM, Weijs PJM. Early high protein intake and mortality in critically ill ICU patients with low skeletal muscle area and –density. Clin Nutr. (2020) 39:2192–201. 10.1016/j.clnu.2019.09.007 31669003

[26] Gomes SM Carvalho C Borges F Ramos A. The impact of early protein intake and nutritional status in critically ill patients. Clin Nutr ESPEN. (2020) 40:412. 10.1016/j.clnesp.2020.09.316

[27] Compher C Chittams J Sammarco T Nicolo M Heyland DK. Greater protein and energy intake may be associated with improved mortality in higher risk critically ill patients: a multicenter, multinational observational study. Crit Care Med. (2017) 45:156–63. 10.1097/CCM.0000000000002083 28098623

[28] Barazzoni R Bischoff SC Breda J Wickramasinghe K Krznaric Z Nitzan D. ESPEN expert statements and practical guidance for nutritional management of individuals with SARS-CoV-2 infection. Clin Nutr. (2020) 39:1631–8. 10.1016/j.clnu.2020.03.022 32305181 PMC7138149

